# Modified expression of alternative oxidase in transgenic tomato and petunia affects the level of *tomato spotted wilt virus *resistance

**DOI:** 10.1186/1472-6750-11-96

**Published:** 2011-10-20

**Authors:** Hao Ma, Congfeng Song, Wayne Borth, Diane Sether, Michael Melzer, John Hu

**Affiliations:** 1 Department of Plant and Environmental Protection Sciences, University of Hawaii, Honolulu, HI 96822, USA; 2 Division of Animal and Nutritional Sciences, West Virginia University, Morgantown, WV 26506, USA; 3 College of Plant Protection, Nanjing Agricultural University, Nanjing, Jiangsu Province 210095, China

## Abstract

**Background:**

*Tomato spotted wilt virus *(TSWV) has a very wide host range, and is transmitted in a persistent manner by several species of thrips. These characteristics make this virus difficult to control. We show here that the over-expression of the mitochondrial alternative oxidase (AOX) in tomato and petunia is related to TSWV resistance.

**Results:**

The open reading frame and full-length sequence of the tomato AOX gene *LeAox1au *were cloned and introduced into tomato 'Healani' and petunia 'Sheer Madness' using *Agrobacterium*-mediated transformation. Highly expressed AOX transgenic tomato and petunia plants were selfed and transgenic R1 seedlings from 10 tomato lines and 12 petunia lines were used for bioassay. For each assayed line, 22 to 32 tomato R1 progeny in three replications and 39 to 128 petunia progeny in 13 replications were challenged with TSWV. Enzyme-Linked Immunosorbent Assays showed that the TSWV levels in transgenic tomato line FKT4-1 was significantly lower than that of wild-type controls after challenge with TSWV. In addition, transgenic petunia line FKP10 showed significantly less lesion number and smaller lesion size than non-transgenic controls after inoculation by TSWV.

**Conclusion:**

In all assayed transgenic tomato lines, a higher percentage of transgenic progeny had lower TSWV levels than non-transgenic plants after challenge with TSWV, and the significantly increased resistant levels of tomato and petunia lines identified in this study indicate that altered expression levels of AOX in tomato and petunia can affect the levels of TSWV resistance.

## Background

Mitochondrial alternative oxidases (AOXs) are important components of the alternative respiratory pathway of plants [1]; Aox genes have been isolated from several important plant species [2-10]. Synthesis of AOX can be induced when the cytochrome pathway is inhibited, or when the plant is wounded, treated with ethylene, cycloheximide, chloramphenicol, or if the plant is exposed to cold environmental conditions [11-15]. In addition, the AOX pathway can also be induced by treatments with salicylic acid (SA) [16], nitric oxide [17], reactive oxygen species [18,19], high light intensities [20] or pathogen challenge. Because SA induction has been linked to the defense response in plants, it has been suggested that the alternative pathway might be associated with disease resistance in plants [21] including resistance to viruses [22,23]. Evidence supporting this hypothesis includes the finding that elevated levels of AOX in tobacco inhibit long-distance movement of *Cucumber mosaic virus *(CMV) and replication of *Tobacco mosaic virus *(TMV) and *Potato virus X *(PVX) [24]. Furthermore, additional works with cytochrome inhibitors and salicylhydroxamic acid (SHAM) have led to the proposal that the AOX pathway and the products of the *Aox *genes play a key role in the resistance of tobacco plants to virus infection [25].

Other studies have suggested, however, that AOX is not a critical component of plant viral resistance but that it may play a role in the development of the hypersensitive response [26]. Elevated *Aox *gene expression levels had no clear-cut effects on SA-induced resistance to systemic infection by TMV in transgenic tobacco. Moreover, resistance to TMV in tobacco induced by antimycin A (AA), an inhibitor of the cytochrome pathway, was repressed with increased alternative pathway capacity, and both SA- and AA-induced resistances were enhanced when alternative pathway capacity was reduced [27,28]. Furthermore, high-levels of alternative oxidase expression allowed increased TMV spread and the development of severe symptoms in NN-type tobacco and *Nicotiana benthamiana *[29]. The involvement of AOX in virus resistance has been reported in a limited number of plant species and virus combinations, however, the mechanisms of this antiviral action varied [3,30,31]. In order to accumulate more evidence that might further elucidate the association of AOX with antiviral activity, we generated transgenic tomato and petunia lines with altered AOX expression levels and evaluated their resistant levels to *tomato spotted wilt virus *(TSWV).

## Results and discussion

### Tomato and petunia transformation and controlled pollination of transgenic lines

**Figure 1:**
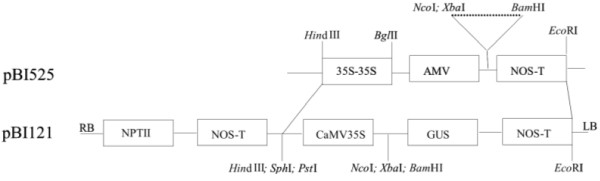
**Constructs developed for plant transformations**. PCR amplicons of the ORF and full-length *LeAox1au *gene were cloned into pBI525 at the *Xba*I and *Bam*HI sites (dashed line). The resulting fragment was excised from pBI525 at the *Hin*d III and *Eco*R I sites and ligated into pBI121 to form the transformation constructs.

**Figure 2:**
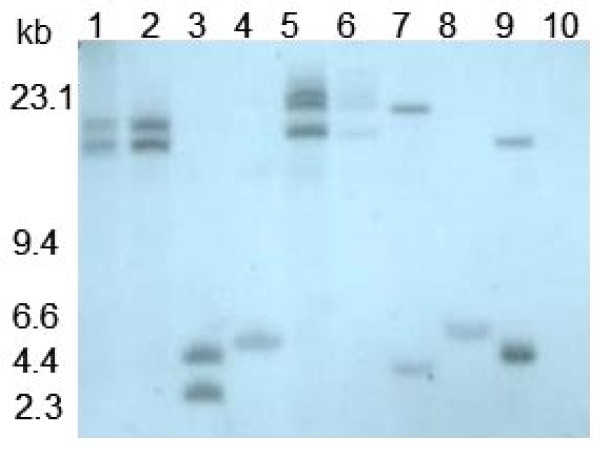
**Southern blot analyses of selected transgenic tomato plants**. Genomic DNA was digested with *Eco*R I, separated in 1% agarose gel, transferred to a nylon membrane, and hybridized to a 620 bp DIG-labeled probe of the 35S promoter sequence. Numbers on the left are approximate sizes in kilobase pairs. Lanes 1 to 10: 1, FDT3-3-1; 2, FDT3-1; 3, CDT1-1; 4, CDT13; 5, CDT17-1; 6, CDT17-3; 7, CKT1; 8, CKT6; 9, 525DT5; 10, wild type.

Tomato cultivar 'Healani' and petunia cultivar 'Sheer Madness' were transformed with the *Leaox*1*au *gene isolated from tomato to obtain transgenic lines. Primers specific for the CaMV 35S promoter and 3'-end of the target inserts pBILaF and pBILaC were used in PCR amplifications to detect the presence of integrated *LeAox1au *sequences. Both wild type and transgenic tomato plants contain the same alternative oxidase gene in their genomes, therefore the CaMV 35S promoter fragment DNA (pBI525 digested with *Hin*d III and *Bgl *II, Figure 1), was used to probe *Eco*RI-digested genomic DNA. A single *Eco*R I recognition site is located near the left border of the inserted sequences, thus each band shown in Figure 2 should represent different insert locations. Tomato lines CDT13 and CKT6 showed single bands, and may represent single copy transformants. The other 7 lines showed 2 to 3 bands, indicating that these 7 lines were transformed with multiple copies of the *LeAox1au *construct.

**Figure 3:**
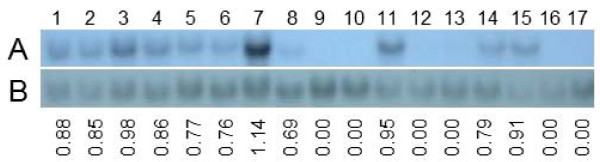
**Northern blot analyses of selected transgenic tomato plants**. Panel A, total RNA prepared from tomato plants was probed with the ORF fragment of *LeAox1au*. Panel B, tomato 18S rRNA. Lanes 1 to 17: 1, FDT3-3-1; 2, FDT3-3-2; 3, FDT3-1; 4, FDT3-3-3; 5, FKT6; 6, FKT7; 7, CDT1-1, 8, CDT6; 9, CDT10; 10, CDT13; 11, CDT16-1; 12, CDT17-1; 13, CDT17-3; 14, CKT1; 15, CKT6; 16, 525DT5; 17, wild type. The values below panel B are the ratios of the intensity of hybridization signal of the sample in panel A verses that in panel B.

**Figure 4:**
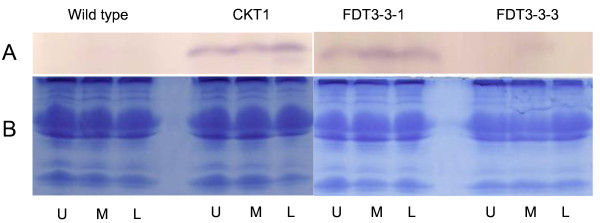
**Western blot analyses of AOX expression of some tomato transgenic and wild type lines**. A, mitochondrial proteins isolated from transgenic lines CKT1, FDT3-3-1, FDT3-3-3 and non-transgenic control plants were separated in 12% SDS-PAGE gels, transferred to PVDF membranes, probed with mouse monoclonal antibody AOA and detected with NBT/BCIP. B, mitochondrial protein stained with Coomassie. U, upper leaf; M, middle leaf; L, lower leaf.

Several putatively transgenic tomato lines were analyzed for *Aox *RNA expression by northern analyses using a *LeAox1-au *specific ORF DNA fragment labeled with DIG as a probe. *Leaox1-au *was not detected in vector only (525DT5) or non-transgenic (wild-type) tomato plants (Figure 3). Expression of *Aox *could not be detected in lines CDT10, CDT13, CDT17-1 and CDT17-3. The remaining eleven transformed lines showed altered expression of *LeAox-1au *ranging from 0.69 to 1.14 times the average signal intensity of the *Aox *ORF verses that of 18S rRNA. No clear relationship between the *Aox *copy number determined by Southern analyses and the *Aox *RNA expression level determined by northern analyses was observed (compare figures 2 and 3). The putative transgenic tomato lines were tested for their AOX protein expression levels by western blot analyses. AOX expression in non-transgenic and vector-only transformed tomato plants was below detection levels (Figure 4). Thirty-two transgenic tomato lines showed increased expression of AOX compared to AOX expression levels in non-transgenic and vector-only transgenic tomato plants. However three transgenic lines (FDT3-3-3, CDT10, and CDT13) did not show increased AOX expression (CDT10 and CDT13 not shown). Based on PCR, Southern, northern, and western blot analyses, a total of 35 tomato and 37 petunia lines were shown to be transformed with the *LeAox1au *gene.

The transgenic lines were self-pollinated and the harvested seeds were dried and stored at 4°C. Seeds of 10 tomato lines and 13 petunia lines were germinated and grown in the greenhouse. When the tomato seedlings reached 4 to 6 cm in height, one leaf disk was collected from each plant using #5 cork borer. Transgene constructs in the progeny were confirmed by PCR and Southern hybridizations. Only confirmed transgenic lines were analyzed further by TSWV challenge.

### Response of R1 generation transgenic tomato plants to TSWV infection

Twenty to 34 R1generation tomato plants from each line were evaluated for resistance to TSWV (Table 1). Within these groups, 17 to 22 lines were confirmed to contain the transgene by PCR analyses. The results showed that within each line, all of the transgenic plants had lower ELISA readings after TSWV challenge than those of non-transgenic plants, indicating that the transgenic tomato plants had elevated resistance level to TSWV infection [32]. Although no lines were found to be completely immune to TSWV, paired t-tests indicated that the progeny from line FKT4-1 showed significantly lower ELISA values than the wild-type control line. Progeny from seven other transgenic lines did not have statistically significant enhanced resistance compared with wild-type controls, which might possibly be related to positional effects of the insertion event or if the transgene interacted somehow with other genes.

**Table 1:** Evaluation of R1-generation of tomato transgenic lines for levels of resistance to TSWV

Line	Total number of R1 plants	Number of plants with AOX transgene	Percentage of transgenic progeny without AOX gene and OD < control	Percentage of transgenic progeny with AOX gene and OD < control	Paired t-test Prob > |T|
CDT9	20	18	50.0	77.8	0.179
FKT12-1	27	20	42.9	60.0	0.597
FKT2	26	17	44.4	76.5	0.076
FKT4-1	32	22	40.0	86.4	0.036*
FKT6	31	22	22.2	72.7	0.095
FKT7	28	19	33.3	68.4	0.257
FKT8	27	19	25.0	57.9	0.892
FKT9	34	17	47.1	82.4	0.254

* indicates significant (P < 0.05) difference

### Response of transgenic tomato plants to TSWV infection at different time points

**Figure 5:**
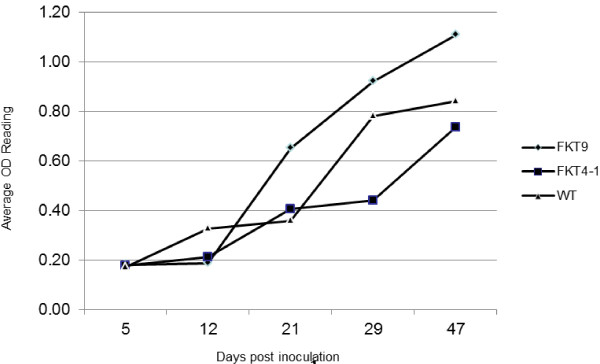
**Mean ELISA readings of progeny from 2 transgenic tomato lines and wild-type control plants**. Two lines of transgenic tomato (FKT9 and FKT4-1) and wild-type control plants were inoculated twice with TSWV and virus titer assayed by ELISA at different time points over a 47 day period.

To evaluate the dynamic response to TSWV infection, 4 to 7 plants of transgenic lines FKT9, FKT4-1, and wild-type controls were evaluated over time after virus challenge. Tissues were sampled and ELISA analyses were performed up to 47 days after the second TSWV inoculation. The data in Figure 5 are the average of three ELISA values from 4-7 plants of each line. Results show that plants of both transgenic lines and the control all developed TSWV infections by 47 days after challenge, which showed small dark spots, bronzed leaves that rolled upward, and dieback of young branches. Line FKT9 was slightly more resistant to TSWV at 12 days post inoculation, but was less resistant to the virus at later times. Plants from line FKT4-1 were more resistant than wild-type control plants at all-time points except at 21 days after inoculation. The resistance of FKT4-1 transgenic progeny over time is consistent with the results presented in Table 1, in which selfed plants from line FKT4-1 that contained the transgene were significantly more resistant to TSWV than selfed plants from this line that did not contain the transgene.

### Response of transgenic petunia plants to TSWV infection

The progeny of 13 transgenic petunia lines were tested for TSWV resistance in a randomized complete block design. In each of the replicates, wild-type non-transgenic controls grown under the same conditions were included among the transgenic lines. Three to four days after TSWV inoculation, necrotic local lesions developed on inoculated leaves of test plants. One transgenic line (FKP10) had significantly smaller local lesion sizes and fewer numbers of local lesions compared with wild-type control plants (Table 2). Twelve other transgenic lines were not significantly different from control plants in their levels of resistance to TSWV infection as measured by the sizes and numbers of local lesions. Because the numbers of R1 petunia plants used in our experiments ranged from 39 to 128 for each line, all of the bioassayed plants were not screened by PCR for the presence of the transgene. However, the data could still indicate a correlation of modified AOX expression in the transgenic progeny with resistance to TSWV infection, even though segregation of AOX expression existed in the R1 population.

**Table 2:** Evaluation of progeny of petunia transgenic line for resistant reaction to TSWV

Line	local lesion diameter	Local lesion numbers
CKP4-1-1	0.264	0.2199
CKP6-6-1	0.3824	0.3528
CKP7-2	0.369	0.3922
CKP8-1	0.4146	0.3103
CKP11-2-1	0.5041	0.3422
CKP15-2-3	0.1324	0.1641
CKP24	0.6186	0.7152
FKP10	0.0458*	0.0384*
FKP16	0.1730	0.1501
FDP1-3-1	0.6076	0.4269
FDP1-6	0.9885	0.9574
FDP2	0.0855	0.0889
FDP14	0.3862	0.2575

The average values of each replication were used for paired t-test of the progenies produced from transgenic petunia lines and wild-type control. The P values in the table were estimated by the replications ranged from 11 to 13.

* indicates significant (P < 0.05) difference.

## Conclusion

Our experiments demonstrate that transgenic tomato line FKT4-1 and transgenic petunia line FKP10, both with elevated AOX expression levels, have higher levels of resistance to TSWV than control plants. These results differ from the reported lack of resistance to tobacco mosaic virus (TMV) in transgenic tobacco with altered levels of AOX [26]. However, in these experiments with tobacco, only two transgenic lines were analyzed. If more transgenic lines had been created and evaluated, different conclusions might have been reached. Several studies have shown that altered AOX activity was positively correlated with resistance of transgenic tobacco and Arabidopsis plants to TMV and CMV infection [23,27,31]. Other, contradictory results were found in TMV challenged tobacco and *N. benthamiana *[29]. As more plant species and viruses have been used to elucidate antiviral mechanisms in plants, it has become clear that different host species can use different mechanisms to resist virus infection [31]. Our results support the hypothesis that the AOX pathway may be associated in some way with plant resistance to viruses. In our experiments and those of others, all plants with modified AOX expression levels that have been evaluated have been challenged with only one virus. It has not been reported how host species with altered AOX levels respond to challenges by different plant viruses. Our transgenic tomato line FKT4-1 and petunia line FKP10 will be challenged with viruses other than TSWV to evaluate their wide-spectrum virus resistance.

## Methods

### Production of transgenic plants

The full-length and ORF only sequences of the *Leaox*1*au *gene isolated from tomato and cloned into pBI525 and subcloned into pBI121 were constructed [9] (Figure 1). Tomato cultivar 'Healani' and petunia cultivar 'Sheer Madness' leaf explants were transformed with these constructs using *Agrobacterium *infection. Total RNAs and plant genomic DNAs were isolated using RNeasy^® ^Plant Mini Kits and DNeasy^® ^Plant Mini Kits (Qiagen, Valencia, CA) respectively. DNA was extracted from selfed R1 plants using a simplified method for screening transgenes [33]. Putatively transformed tomato and petunia plants and the progeny of selfed primary transgenic lines were screened by PCR using 35S-specific primer pairs (5'-GACATCTCCACTGACGTAAGG-3' and 5'-CTCAACACATGAGCGAAACC-3') or (35SF: 5'-AAAGGAAGGTGGCTCCTACAAAT-3' and 35SR: 5'-CTCTCCAAATGAAATGAAATGAACTTCC-3') [34]. DNA and RNA hybridizations, electrophoresis, and blotting were done according to Sambrook and Russell (2001)[35]. Chemiluminescent detection was conducted using the DIG High-Prime DNA Labeling and Detection Starter Kit II^® ^(Roche, Indianapolis, IN). The 35S probe was prepared by PCR with plasmid PBI525 DNA as template, and the 18SrDNA control probe was amplified by PCR (18SF:5'-CCTCAGAAACCGCTACCAC-3' and 18SR: 5'-AATACGAATCCCCCCGAC-3') using genomic DNA as template. Both probes were purified with the Concert^® ^PCR purification system (Life Technologies, Grand Island, NY). Band intensities in northern blot analyses were measured using a Bio-Rad Discovery Series Quantity One^® ^image analyzer and software (Bio-Rad,Hercules,CA).

For western blot analyses, mitochondrial proteins were extracted according to Boutry et al. [36] with modifications. Briefly, 0.1 g plant leaves were ground in 1 ml extraction buffer (0.4 M sucrose, 50 mM Tris base,1 mM EGTA, 5 mM 2-mercaptoethanol, 1% bovine serum albumin, 10 mM KH_2_PO_4_, 0.1% polyvinylpolypyrrolidone, pH 7.6) and the homogenate was filtered through 4 layers of Miracloth^® ^(Calbiochem, La Jolla, CA). The filtrate was centrifuged at 3000 g for 10 minutes in a Sorvall SS34 rotor and the supernatant was then centrifuged at 25,000 g for 10 min in the same rotor. The resulting pellet containing mitochondria was dissolved in 50 μl suspension buffer (0.4 M mannitol, 0.5% bovine serum albumin, 10 mM KH_2_PO_4_, pH 7.2), and sample aliquots (5 μl) were analyzed by electrophoresis in 12% SDS-PAGE gels. Gels were stained with Comassie Brilliant Blue R-250 according to Sambrook & Russell (2001) and protein fragments were transferred onto PVDF membrane by electroblotting. Detection of AOX protein was done using the mouse monoclonal antibody Alternative Oxidase All (AOA) raised against *Sauromatum guttatum *AOX [37].

The confirmed transgenic tomato and petunia lines were selfed and seeds of 24 transgenic tomato lines and 33 transgenic petunia lines were collected. Ten selfed transgenic tomato lines and 1 non-transgenic control tomato were challenged with TSWV by mechanical inoculation with three replications. Segregated transgenic and non-transgenic plants were identified by PCR. Twenty-two to 32 tomato R1 transgenic plants from each line were confirmed by PCR, challenged with TSWV, and analyzed by enzyme-linked immunosorbent assay (ELISA). For petunia R1 plants, 13 replications each with about 10 plants were used for TSWV screening in a randomized complete block design.

### TSWV infection and plant evaluation

TSWV was isolated from a tomato plant with typical symptoms of TSWV infection (small dark spots on leaves, bronzed leaves that rolled upward, and dieback of young branches) grown on a farm on Oahu, Hawaii. When R1 tomato seedlings had grown 4 to 6 cm height, the individual plants were transplanted into single pots and grown to the 5 - 6 leaf stage. These plants were then grown at 22 to 25°C under 16/8 hr. photoperiod before virus challenge. On one fully-expanded young leaf of each plant, five carborundum-dusted leaflets were inoculated with 100 μl freshly-prepared TSWV inoculum made by grinding tomato leaves systemically-infected with TSWV in phosphate buffer (0.033 M KH_2_PO_4_, 0.067 M Na_2_HPO_4_, pH7.0**) (**1:10, w/v) supplemented with 10 mM sodium sulfite. All TSWV extracts were kept on ice until all plants had been inoculated. Seven to ten days after the first inoculation, all the plants were inoculated for a second time as above. About 20 to 30 days after the second inoculation, Immunostrips^® ^(Agdia, Elkhart Ind.) were used to assay challenged plants for TSWV infection. If positive plants were confirmed, then fully-expanded new leaves were collected for ELISA [38]. The absorbance values at 405 nm were determined in a microplate reader (Bio-Rad model 680, Hercules, CA). For petunia seedlings, within each replicate, randomly selected plants were dusted with carborundum on three young leaves. Fifty microliters of fresh TSWV inoculum, prepared as above was inoculated onto each of three leaves on each plant. Four to seven days after inoculation, the number and diameters of local lesions were recorded.

### Data analysis

For each assayed tomato sample, extracts from three leaf disks prepared as above were collected and analyzed in adjacent wells of ELISA plates. A sample was considered positive if the average absorbance value of the three replicate wells was four times greater than the average absorbance value of healthy uninoculated samples of non-transgenic plants analyzed in the same plate [39]. Samples from R1 transgenic tomato plants and wild-type controls from each plate, and petunia plants and wild-type controls from each replicate were considered paired values to conduct two-sample paired t-tests with SAS^® ^software.

## Authors' contributions

HM and CS: Carried out cloning, transformation, tissue culture, characterization of transgenic lines, plant pollination and propagation, bioassay, data analysis, and drafted the manuscript; DS and MM provided technical support; WB and JH contributed overall project design and were the project leaders. All authors read and approved the final manuscript.

## References

[B1] Van Aken O Giraud E Clifton R Whelan J Alternative oxidase: a target and regulator of stress responses Physiol Plantarum 2009 137 4 354 361 10.1111/j.1399-3054.2009.01240.x 19470093

[B2] Kumar AM Soll D Arabidopsis alternative oxidase sustains Escherichia coli respiration Proc Natl Acad Sci USA 1992 89 22 10842 10846 10.1073/pnas.89.22.10842 PMC50438 1438286

[B3] Vanlerberghe GC McIntosh L Mitochodrial electron transport regulaton of nuclear gene expression - Studies with the alternative oxidase gene of tobacco Plant Physiol 1994 105 8 10.1104/pp.105.3.867 PMC160734 8058837

[B4] Cruzhernandez A Gomezlim MA Alternative Oxidase from Mango (Mangifera-Indica, L) Is Differentially Regulated during Fruit Ripening Planta 1995 197 4 569 576 10.1007/BF00191562 8555961

[B5] Hiser C Kapranov P McIntosh L Genetic modification of respiratory capacity in potato Plant Physiology 1996 110 1 277 286 10.1104/pp.110.1.277 PMC157719 8587988

[B6] Whelan J Millar AH Day DA The alternative oxidase is encoded in a multigene family in soybean Planta 1996 198 2 197 201 10.1007/BF00206244 8580775

[B7] Ito Y Saisho D Nakazono M Tsutsumi N Hirai A Transcript levels of tandem-arranged alternative oxidase genes in rice are increased by low temperature Gene 1997 203 2 121 129 10.1016/s0378-1119(97)00502-7 9426242

[B8] Holtzapffel RC Castelli J Finnegan PM Millar AH Whelan J Day DA A tomato alternative oxidase protein with altered regulatory properties Bba-Bioenergetics 2003 1606 1-3 153 162 10.1016/s0005-2728(03)00112-9 14507436

[B9] Song CF Borth W Wang JS Hu JS Cloning and expression of an alternative oxidase gene from Lycopersicon esculentum Zhi Wu Sheng Li Yu Fen Zi Sheng Wu Xue Xue Bao 2004 30 5 503 510 15627703

[B10] Cardoso HG Campos MD Costa AR Campos MC Nothnagel T Arnholdt-Schmitt B Carrot alternative oxidase gene AOX2a demonstrates allelic and genotypic polymorphisms in intron 3 Physiol Plantarum 2009 137 4 592 608 10.1111/j.1399-3054.2009.01299.x 19941625

[B11] Morohashi Y Seto T Matsushima H Appearance of Alternative Respiration in Cucumber Cotyledon Mitochondria after Treatment with Cycloheximide Physiol Plantarum 1991 83 4 640 646

[B12] McIntosh L Molecular biology of the alternative oxidase Plant Physiol 1994 105 3 781 786 10.1104/pp.105.3.781 PMC160724 8058835

[B13] Day DA Whelan J Millar AH Siedow JN Wiskich JT Regulation of the Alternative Oxidase in Plants and Fungi Aust J Plant Physiol 1995 22 3 497 509

[B14] Zhang QS Mischis L Wiskich JT Respiratory responses of pea and wheat seedlings to chloramphenicol treatment Aust J Plant Physiol 1996 23 5 583 592

[B15] Searle SY Thomas S Griffin KL Horton T Kornfeld A Yakir D Hurry V Turnbull MH Leaf respiration and alternative oxidase in field-grown alpine grasses respond to natural changes in temperature and light New Phytol 2011 189 4 1027 1039 10.1111/j.1469-8137.2010.03557.x 21128944

[B16] Kapulnik Y Yalpani N Raskin I Salicylic Acid induces cyanide-resistant respiration in tobacco cell-suspension cultures Plant Physiol 1992 100 4 1921 1926 10.1104/pp.100.4.1921 PMC1075885 16653218

[B17] Huang X von Rad U Durner J Nitric oxide induces transcriptional activation of the nitric oxide-tolerant alternative oxidase in Arabidopsis suspension cells Planta 2002 215 6 914 923 10.1007/s00425-002-0828-z 12355151

[B18] Costa JH Mota EF Cambursano MV Lauxmann MA de Oliveira LM Silva Lima Mda G Orellano EG Fernandes de Melo D Stress-induced co-expression of two alternative oxidase (VuAox1 and 2b) genes in Vigna unguiculata J Plant Physiol 2010 167 7 561 570 10.1016/j.jplph.2009.11.001 20005596

[B19] Eprintsev AT Mal'tseva EV Shatskikh AS Popov VN [Involvement of hydrogen peroxide in the regulation of coexpression of alternative oxidase and rotenone-insensitive NADH dehydrogenase in tomato leaves and calluses] Izv Akad Nauk Ser Biol 2011 1 45 51 21442906

[B20] Dinakar C Raghavendra AS Padmasree K Importance of AOX pathway in optimizing photosynthesis under high light stress: role of pyruvate and malate in activating AOX Physiol Plant 2010 139 1 13 26 10.1111/j.1399-3054.2010.01346.x 20059739

[B21] Simons BH Millenaar FF Mulder L Van Loon LC Lambers H Enhanced expression and activation of the alternative oxidase during infection of Arabidopsis with Pseudomonas syringae pv tomato Plant Physiology 1999 120 2 529 538 10.1104/pp.120.2.529 PMC59291 10364404

[B22] Chivasa S Murphy AM Naylor M Carr JP Salicylic Acid Interferes with Tobacco Mosaic Virus Replication via a Novel Salicylhydroxamic Acid-Sensitive Mechanism Plant Cell 1997 9 4 547 557 10.1105/tpc.9.4.547 PMC156938 12237364

[B23] Chivasa S Carr JP Cyanide restores N gene-mediated resistance to tobacco mosaic virus in transgenic tobacco expressing salicylic acid hydroxylase Plant Cell 1998 10 9 1489 1498 10.1105/tpc.10.9.1489 PMC144082 9724695

[B24] Naylor M Murphy AM Berry JO Carr JP Salicylic acid can induce resistance to plant virus movement Mol Plant Microbe In 1998 11 9 860 868

[B25] Murphy AM Chivasa S Singh DP Carr JP Salicylic acid-induced resistance to viruses and other pathogens: a parting of the ways? Trends Plant Sci 1999 4 4 155 160 10.1016/s1360-1385(99)01390-4 10322550

[B26] Ordog SH Higgins VJ Vanlerberghe GC Mitochondrial alternative oxidase is not a critical component of plant viral resistance but may play a role in the hypersensitive response Plant Physiol 2002 129 4 1858 1865 10.1104/pp.003855 PMC166774 12177499

[B27] Gilliland A Singh DP Hayward JM Moore CA Murphy AM York CJ Slator J Carr JP Genetic modification of alternative respiration has differential effects on antimycin A-induced versus salicylic acid-induced resistance to Tobacco mosaic virus Plant Physiology 2003 132 3 1518 1528 10.1104/pp.102.017640 PMC167090 12857832

[B28] Singh DP Moore CA Gilliland A Carr JP Activation of multiple antiviral defence mechanisms by salicylic acid Mol Plant Pathol 2004 5 1 57 63 10.1111/j.1364-3703.2004.00203.x 20565582

[B29] Murphy AM Gilliland A York CJ Hyman B Carr JP High-level expression of alternative oxidase protein sequences enhances the spread of viral vectors in resistant and susceptible plants J Gen Virol 2004 85 Pt 12 3777 3786 10.1099/vir.0.80385-0 15557251

[B30] Robson CA Vanlerberghe GC Transgenic plant cells lacking mitochondrial alternative oxidase have increased susceptibility to mitochondria-dependent and -independent pathways of programmed cell death Plant Physiol 2002 129 4 1908 1920 10.1104/pp.004853 PMC166780 12177505

[B31] Mayers CN Lee KC Moore CA Wong SM Carr JP Salicylic acid-induced resistance to Cucumber mosaic virus in squash and Arabidopsis thaliana: contrasting mechanisms of induction and antiviral action Mol Plant Microbe Interact 2005 18 5 428 434 10.1094/MPMI-18-0428 15915641

[B32] Ndjiondjop MN Albar L Fargette D Fauquet C Ghesquiere A The genetic basis of high resistance to rice yellow mottle virus (RYMV) in cultivars of two cultivated rice species Plant Dis 1999 83 10 931 935 10.1094/PDIS.1999.83.10.931 30841075

[B33] Xin ZG Velten JP Oliver MJ Burke JJ High-throughput DNA extraction method suitable for PCR Biotechniques 2003 34 4 820 + 10.2144/03344rr04 12703307

[B34] Frary A Earle ED An examination of factors affecting the efficiency of Agrobacterium-mediated transformation of tomato Plant Cell Rep 1996 16 3-4 235 240 10.1007/BF01890875 24177560

[B35] Sambrook J Russell DW (eds) Molecular Cloning: A Laboratory Manual 2001 Cold Spring Harbor Laboratory, New York

[B36] Boutry M Briquet M Mitochondrial Modifications Associated with the Cytoplasmic Male-Sterility in Faba Beans Eur J Biochem 1982 127 1 129 135 10.1111/j.1432-1033.1982.tb06846.x 6890454

[B37] Elthon TE Nickels RL McIntosh L Monoclonal antibodies to the alternative oxidase of higher plant mitochondria Plant Physiol 1989 89 4 1311 1317 10.1104/pp.89.4.1311 PMC1056014 16666702

[B38] Wu ZC Hu JS Polston JE Ullman DE Hiebert E Complete nucleotide sequence of a nonvector-transmissible strain of Abutilon mosaic geminivirus in Hawaii Phytopathology 1996 86 6 608 613

[B39] Stevens MR Scott ST Gergerich RC Evaluation of seven Lycopersicon species for resistance to tomato spotted wilt virus (TSWV) Euphytica 1994 80 6

